# Mesenchymal Stem Cells with Simultaneous Overexpression of GPX3 and CD47 for the Treatment of Drug-Induced Acute Liver Injury

**DOI:** 10.3390/vetsci12020149

**Published:** 2025-02-10

**Authors:** Yuanxiang Jing, Balun Li, Aili Aierken, Zengyu Zhang, Dongyao Han, Zixi Lin, Jiaqi Gao, Hongkai Tian, Jinlian Hua

**Affiliations:** 1 College of Veterinary Medicine, Shanxi Centre of Stem Cells Engineering & Technology, Northwest A&F University, Yangling 712100, China; jingyuanxiang@outlook.com (Y.J.); libalun@nwafu.edu.cn (B.L.); alijan@xjmu.edu.cn (A.A.); 2018010993@nwafu.edu.cn (Z.Z.); handongyaoyao@163.com (D.H.); 16602914351@163.com (Z.L.); gjq1223@126.com (J.G.); thk20090601@163.com (H.T.); 2 Key Laboratory of Pathogenesis, Prevention and Treatment of High Incidence Diseases in Central Asia, Xinjiang Medical University, Urumqi 830000, China

**Keywords:** mesenchymal stem cells (MSCs), GPX3, CD47, drug-induced liver injury

## Abstract

The liver, as the largest metabolic and detoxification organ in mammals, metabolizes approximately 80–90% of drugs. However, drug-induced liver injury (DILI) is common and driven by factors such as individual variability, differences in liver metabolism, and improper drug use. Mesenchymal stem cells (MSCs), with their self-renewal and multipotent differentiation capabilities, offer therapeutic potential, but face challenges such as limited proliferation and increased apoptosis during in vitro expansion. Although MSCs exhibit low immunogenicity, they are often cleared by the host immune system, which limits their survival and engraftment. Glutathione peroxidase 3 (GPX3) is a key antioxidant enzyme that reduces reactive oxygen species (ROS), protecting cells from oxidative damage. CD47, also known as integrin-associated protein (IAP), helps cells evade immune clearance by binding to signal regulatory protein alpha (SIRPα) on the immune cells. Here, we used an acetaminophen (APAP)-induced DILI mouse model to evaluate the therapeutic efficacy of intravenously infused MSCs overexpressing GPX3 and CD47. Compared to unmodified MSCs, modified MSCs showed improved survival, reduced liver inflammation, and alleviated oxidative damage, offering enhanced protection against APAP-induced DILI.

## Simple Summary

In veterinary clinics, drug-induced liver injury caused by toxicity or immune reactions is increasingly being observed. Severe drug-induced liver damage can lead to acute liver failure and death. In this study, we used transplanted mesenchymal stem cells overexpressing exogenous genes to alleviate drug-induced liver injury in mice and dogs. The results showed that the transplantation of mesenchymal stem cells overexpressing exogenous genes significantly reduced inflammation and apoptosis levels in the liver, promoted the proliferation ability of liver cells, thus alleviating liver damage.

## 1. Introduction

Drug-induced liver injury (DILI) is liver damage caused by drugs or other chemical substances, and is one of the most common adverse drug reactions in clinical practice [1]. A wide range of drugs can induce DILI through complex mechanisms. In recent years, significant progress has been made in the diagnosis, including pathological mechanism, and treatment of DILI through clinical practice and basic research [2]. Common drugs leading to DILI include prescription medications, over-the-counter drugs, herbal medicines, and dietary supplements. Among them, prescription drugs, such as acetaminophen, antibiotics (e.g., amoxicillin-clavulanate), antiepileptic drugs (e.g., phenytoin), and non-steroidal anti-inflammatory drugs, are the most frequent cause [3,4,5,6]. These drugs can cause liver damage via various mechanisms [7]. For instance, N-acetyl-p-benzoquinone imine (NAPQI), the primary toxic metabolite of acetaminophen produced during liver metabolism [8], causes hepatocyte injury via oxidative stress, apoptosis, and necrosis [9,10,11].

Currently, treatments for DILI mainly include antioxidant and anti-inflammatory drugs, immunosuppressants, supportive therapies, and early gastrointestinal decontamination. Antioxidant drugs, such as vitamin C and S-adenosylmethionine (SAMe), can alleviate oxidative damage in the liver, reduce reactive oxygen species (ROS) levels, and reduce inflammation [12]. SAMe promotes hepatocyte repair and regeneration and reduces liver inflammation and fibrosis. Clinically, SAMe has been used to treat liver injuries induced by various drugs, such as antidepressants and antineoplastic agents [13]. Anti-inflammatory drugs and immunosuppressants play important roles in treatment [14]. Studies have shown that corticosteroids (e.g., prednisone) are effective in treating drug-induced autoimmune hepatitis, significantly reducing liver inflammation and necrosis [15,16], although concerns remain regarding the long-term safety of corticosteroids in treating DILI [17,18]. Supportive therapy is a critical component of DILI treatment, which involves intravenous infusion of electrolytes and glucose to maintain fluid and electrolyte balance. However, these therapies cannot reverse severe liver damage or promote hepatocyte regeneration, thus presenting limitations for clinical applications.

In recent years, an increasing number of studies have demonstrated the significant potential of stem cells, particularly mesenchymal stem cells (MSCs), for tissue repair and regeneration [19,20,21,22,23,24,25]. MSCs are a typical type of adult stem cell with self-renewal and directed differentiation capabilities [26,27] and are found in various tissues [28,29]. In the treatment of liver injury, MSCs can effectively differentiate into cells with hepatocyte function under specific induction conditions [30,31,32], thereby replacing damaged hepatocytes and restoring liver function [33,34]. Research has shown that, in liver injury models, MSCs can successfully differentiate into cells expressing hepatocyte-specific markers (such as ALB and AFP) under specific induced culture conditions [35]. These differentiated hepatocyte-like cells not only perform metabolic functions but also secrete key liver proteins such as albumin [36]. Furthermore, MSCs exert paracrine effects by secreting a variety of growth factors, cytokines, and other bioactive molecules, thereby promoting tissue repair [37]. For instance, vascular endothelial growth factor (VEGF) secreted by MSCs promotes angiogenesis and improves the blood supply to damaged tissues, thereby facilitating tissue repair [38].

However, mesenchymal stem cells (MSCs) undergo replicative senescence during prolonged in vitro culture, resulting in a reduction in their proliferative capacity and differentiation potential [39,40]. Research has shown that MSCs retain a high differentiation capacity during early passages, but as the passage number increases, their potential to differentiate into osteocytes, chondrocytes, and adipocytes gradually diminishes [41,42]. Additionally, after MSC transplantation into the host, they may be recognized and eliminated by the host immune system. For instance, MSCs can be targeted by host natural killer (NK) cells, which recognize the low expression of major histocompatibility complex (MHC) class I molecules and other activation markers on MSCs, triggering a cytotoxic response that results in the clearance of transplanted cells [43]. This phenomenon is particularly pronounced in the early stages of transplantation and adversely affects MSCs’ survival and function.

Given the aforementioned limitations of MSCs in clinical applications, we simultaneously overexpressed GPX3 and CD47 genes in MSCs using lentiviral transduction to enhance their therapeutic potential and evaluate their efficacy in a DILI model. Glutathione peroxidase 3 (GPX3) is a key antioxidant enzyme that scavenges reactive oxygen species (ROS), protecting cells from oxidative damage [44,45]. CD47, also known as integrin-associated protein (IAP), is a widely expressed membrane protein that binds specifically to signal regulatory protein alpha (SIRPα) on immune cells [46], thereby helping cells evade phagocytosis [47]. Upregulation of these two genes in MSCs aims to resolve issues of host immune rejection, while also enhancing the antioxidant capacity and ability of MSCs to survive in vivo [48,49]. This strategy offers a novel approach for clinical treatment of DILI.

## 2. Materials and Methods

### 2.1. Preparation and Establishment of Cells

All the MSCs described below are canine adipose mesenchymal stem cells, which were isolated, amplified, and characterized in the previous stage of our laboratory [50,51,52,53].

RNA was isolated from canine lung and kidney and reverse transcribed into cDNA. The GPX3 and CD47 gene fragments were amplified by PCR, and the vector was linearized by double enzyme digestion. Homologous recombination was used to link the vectors and the target fragment. MSC/GPX3&CD47 were obtained by lentivirus transfection and continuous screening. The operation details can be found in the Supplemental Materials File S1.

### 2.2. In Vitro Co-Culture Experiments

First, the MSC/GPX3 and MSC/GPX3&CD47 cells cultured to the logarithmic growth phase were stained with PKH 26. The procedure was as follows: the cells were digested with trypsin, the trypsin was discarded after centrifugation, and the cell suspension was resuspended in α-MEM medium. PKH26 dye was diluted with dilution C in a certain ratio, usually 1:250–1:500, according to the manufacturer’s instructions. The mixture was homogeneous to avoid bubble formation and 5 to 10 times the volume of α-MEM was added immediately after the end of the staining time to terminate the staining reaction. Excess staining solution was removed by centrifugation, the cells were re-suspended in a new culture dish for culture, and RWA 264.7 cells were added to the stained MSCs at a ratio of 1:1 and cultured together [54]. The medium was changed daily, and the cell status was observed under fluorescence.

### 2.3. Establishment of Canine and Mouse DILI Model

To further assess the promoting effect of MSC/GPX3&CD47 on DILI, a drug-induced liver injury model was established by intraperitoneal injection of 200 mg/kg APAP in mice [55] and dogs, as described in the Supplemental Materials File S1. Twenty-four hours after modeling, cells were administered via tail vein injection at a dose of 1 × 10^6^ cells per mouse [56]. For dogs, cells were injected into the subcutaneous vein of the forearm at a dose of 1 × 10^7^ cells per dog [57,58,59]. On day 14 post-injection, experimental animals were euthanized, and tissue and blood samples were collected for subsequent analysis. The animals were randomly assigned to groups using computer-generated random numbers. All animal procedures were performed in accordance with the ARRIVE guidelines and all experimental protocols were carried out according to the guidelines established by the Chinese National Standard GB/T35892-2018 (guidelines for the ethical review of laboratory animal welfare), submitted, and previously approved by the Ethics Committee on the Use of Animals of the Northwest A&F University (approval number: NWAFU.No20234580d0600601[200]).

### 2.4. Histology, Immunohistochemistry Staining Evaluation

Liver and blood samples were collected on the last day of treatment. Hematoxylin-eosin (HE) staining was used to evaluate histological changes in the liver. Masson staining was used to evaluate the collagen deposition in the wound bed. PAS staining was used to assess glycogen deposition in the liver. Immunohistochemical staining was performed to detect the expression of TNF-α, BCL-2, PCNA, CYP2E1, and GPX3.

### 2.5. RNA Sequencing

On day 7, mouse livers were collected for total RNA extraction, and RNA sequencing was performed using the HiSeq 2500 (Illumina) system. Genes with a *p* value < 0.05 and fold change > 1.0, were considered significant differential genes.

### 2.6. Statistical Analysis

All experimental data were statistically analyzed, and the results were shown as mean ± standard deviation (SD). Statistical differences were determined by one-way ANOVA and a student-*t*-test. In all cases, if *p* < 0.05, there is a significant difference.

## 3. Results

### 3.1. Establishment and Biological Characteristics of MSC/GPX3&CD47

#### 3.1.1. Establishment of MSC/GPX3&CD47

**Figure 1:**
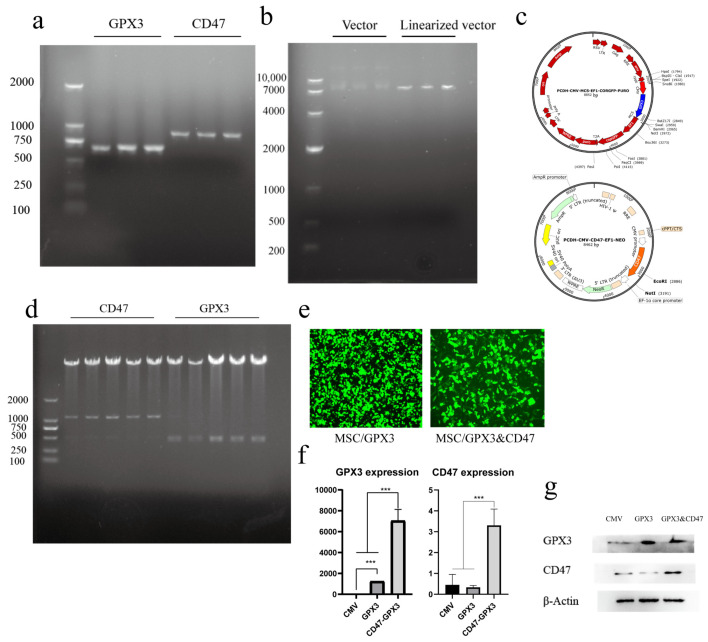
Establishment of MSC/GPX3&CD47. (**a**) PCR amplification of GPX3 and CD47 gene fragments; (**b**) result of vector linearization; (**c**) vector map; (**d**) double enzyme digestion identification results; (**e**) lentiviral transduction of MSCs; (**f**) result of qRT-PCR; (**g**) result of Western blot. (*** *p* ≤ 0.001).

In this study, we integrated exogenous genes into a lentiviral vector via homologous recombination and transfected them into MSCs using lentiviral transduction, allowing integration into the MSC genome. MSCs/GPX3 and CD47 were obtained after repeated selection with puromycin and G418, respectively. The target genes were fragments obtained by PCR, with GPX3 having a length of 681 bp and CD47 having a length of 912 bp (Figure 1a). The vector was linearized (Figure 1b) and homologous recombination was used to link the target fragments with the vector. Figure 1c shows a map of successfully ligated vectors. Double-enzyme digestion was performed for validation, and the results confirmed that the target fragments were successfully ligated into the vector (Figure 1d). Figure 1e shows successful transfection of MSCs with the lentiviral vector carrying the target fragments. RNA and protein were collected from the cells for analysis, and Figure 1f,g show that both mRNA and protein expression levels were significantly increased. In conclusion, we successfully constructed an MSC line overexpressing GPX3 and CD47.

#### 3.1.2. RNA-Seq Differential Gene Expression Analysis

**Figure 2:**
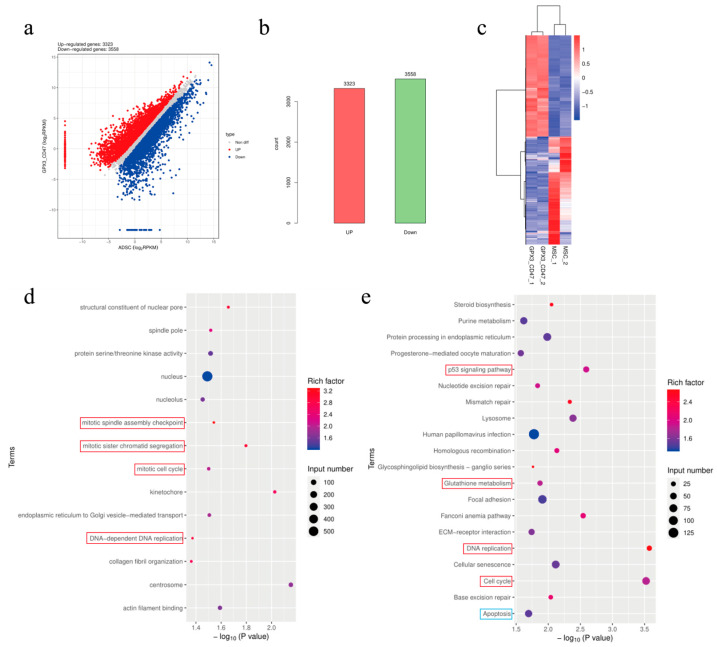
Differential gene analysis between MSC and MSC/GPX3&CD47 groups. (**a**) Volcano plot; (**b**) DEG counts; (**c**) heatmap of differential genes; (**d**) GO enrichment analysis; (**e**) KEGG enrichment analysis.

The volcano plot of differentially expressed genes showed that, compared to the gene expression in MSCs, 3323 genes were upregulated and 3558 genes were downregulated in MSC/GPX3&CD47 (Figure 2a,b). The heatmap of gene expression further illustrates the trends in gene expression changes (Figure 2c). GO enrichment analysis revealed significant upregulation of differentially expressed genes related to the mitotic spindle assembly checkpoint, mitotic sister chromatid segregation, mitotic cell cycle, and DNA-dependent DNA replication pathways in MSC/GPX3&CD47 cells (Figure 2d). The enrichment of these pathways suggests that GPX3 and CD47 overexpression promotes cell proliferation. KEGG enrichment analysis (Figure 2e) showed that the differentially expressed genes were enriched in the p53 signaling pathway, a critical tumor suppression pathway involved in regulating cell cycle, DNA repair, senescence, and apoptosis. Enrichment of this pathway indicates improved cell signaling, potentially involving increased resistance to oxidative stress and modifications in the cell cycle. The glutathione metabolism pathway was also enriched, indicating that the activation of glutathione metabolism helps cells repair damaged proteins, lipids, and DNA. Glutathione also maintains the integrity of the protein structure and function by regulating the redox state of thiol groups, protecting cell membrane stability. Enrichment of the Wnt signaling pathway suggests the regulation of processes related to cell proliferation and differentiation, which may affect the developmental state or differentiation pathways of cells. Cell cycle and DNA replication pathways were also significantly upregulated, indicating a marked increase in cell proliferation capacity. Additionally, downregulation of the apoptosis pathway suggested that the overexpression of GPX3 and CD47 reduced the level of apoptosis.

GO and KEGG analyses demonstrated that gene modification promotes the mitotic spindle pathway, DNA replication, and cell cycle regulation. Simultaneously, the activation of the p53 signaling pathway, glutathione metabolism, and downregulation of apoptosis-related genes indicated that the overexpression of GPX3 and CD47 enhanced DNA replication, cell proliferation, and antioxidant capabilities of MSCs while inhibiting apoptosis-related gene expression.

#### 3.1.3. In Vitro Biological Characteristics of MSC/GPX3&CD47

In this section, we simulated the immune phagocytosis process of MSCs in vivo by co-culturing them with murine macrophages in vitro [60] to investigate the characteristics of mesenchymal stem cells (MSCs) co-expressing GPX3 and CD47 in vitro. RAW 264.7, a macrophage cell line derived from mice, was initially isolated from mouse leukemia cells induced by the Abelson leukemia virus and is widely used in studies of immunology, inflammation, and cancer [61]. RAW 264.7 cells possess typical macrophage characteristics, including phagocytosis, cytokine production, and immune response. GPX3 is an antioxidant enzyme that helps cells resist oxidative stress, whereas CD47 is known as a “do not eat me” signal molecule that inhibits macrophage phagocytosis by interacting with signal regulatory protein alpha (SIRPα) on macrophages. By co-expressing these two genes, we aimed to improve the survival rate of MSCs in vivo, reduce the likelihood of them being cleared by the host immune system, and thereby enhance their therapeutic efficacy.

**Figure 3:**
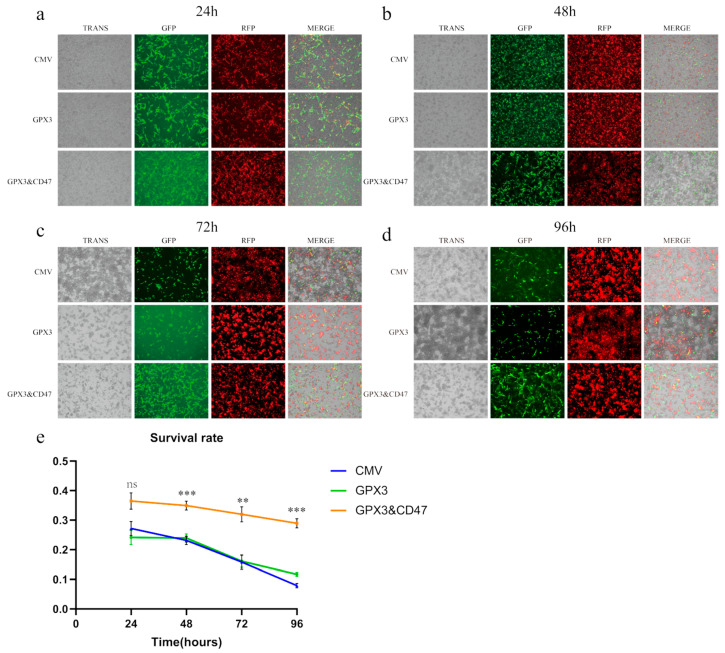
Co-culture of MSCs and RAW 264.7 cells in vitro (100× magnification). (**a**) 24 h; (**b**) 48 h; (**c**) 72 h; (**d**) 96 h; (**e**) cell survival rate. (ns *p* > 0.05, ** *p* ≤ 0.01, *** *p* ≤ 0.001).

First, we co-cultured MSCs with RAW264.7 macrophages to observe the phagocytosis of MSCs by macrophages. We analyzed the survival rate of MSCs at different time points. MSCs transduced with PCDH-CMV empty vector were used as controls, hereafter referred to as MSC/CMV or CMV. All cells were stained with PKH 26 dye to turn the cell membranes red before co-culture. The CMV group consisted of MSCs carrying an empty vector, while the GPX3 group and the GPX3&CD47 group were MSCs overexpressing GPX3 and MSCs overexpressing both GPX3 and CD47, respectively. All groups of MSCs were labeled with GFP green fluorescence. As shown in the figure (Figure 3), after 24 h of co-culture with RAW cells, the survival rate of MSC/CMV and MSC/GPX3 cells was approximately 25%, while the survival rate of MSC/GPX3 and CD47 cells, which overexpress CD47, was 36.1%. Within 24 h, most MSCs were cleared by RAW cells, but the survival rate of CD47-overexpressing cells was slightly higher than that of the other cells.

At 96 h, cell observation and survival rate analysis showed that MSC/GPX3 and CD47 had a significantly higher survival rate than the other two groups. By 96 h, more than 90% of MSC/CMV cells had been cleared and more than 85% of MSC/GPX3 cells had died. These results suggest that the overexpression of CD47 enhanced the immune evasion ability of MSCs to some extent, resulting in fewer cells being cleared by macrophages in co-culture with RAW 264.7 macrophages.

### 3.2. MSC/GPX3&CD47 Promote Proliferation, Reduce Inflammatory Responses, and Prevent Apoptosis to Rescue APAP-Induced Liver Injury

In this study, mouse and canine DILI models induced by APAP were constructed. Canine adipose-derived mesenchymal stem cells co-expressing GPX3 and CD47 were intravenously infused and tissue and blood samples were collected 14 days later. The therapeutic effect of MSCs co-expressing GPX3 and CD47 on DILI was observed using histopathology, biochemical indicators, immunohistochemistry staining, and transcriptome sequencing.

**Figure 4:**
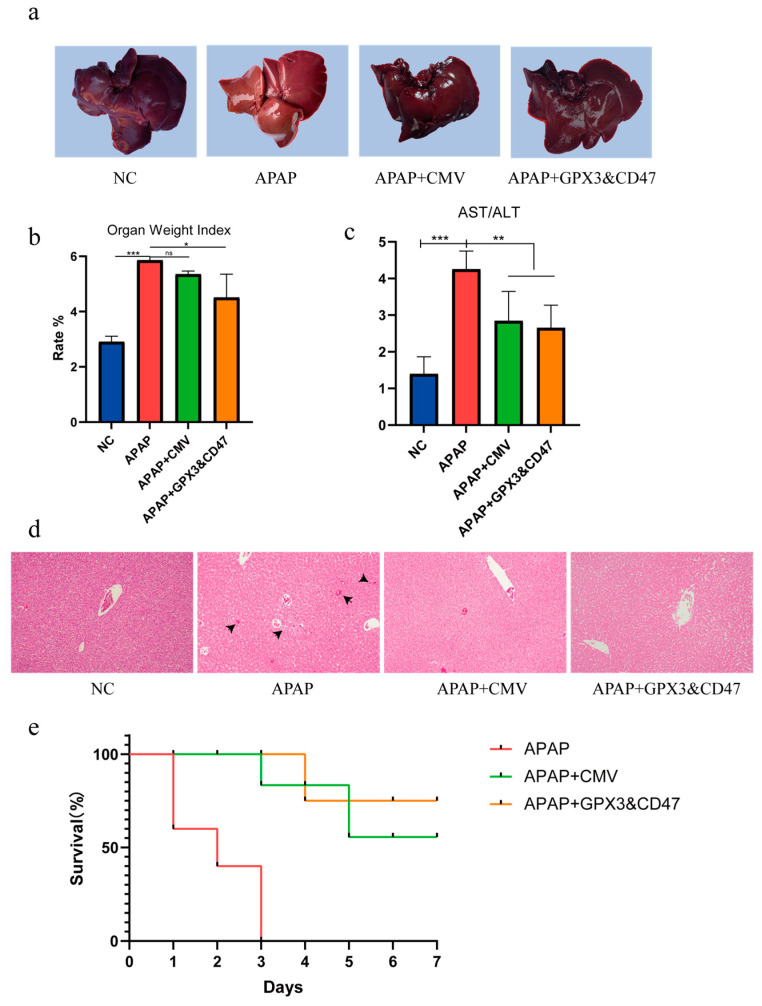
Therapeutic effects of MSC/GPX3&CD47 on canine DILI model. (**a**) Canine liver necropsy; (**b**) Liver Organ Index; (**c**) liver function results; (**d**) liver HE staining (200× magnification); (**e**) canine survival curve. (ns *p* > 0.05, * *p* ≤ 0.05, ** *p* ≤ 0.01, *** *p* ≤ 0.001).

First, we established a DILI model in dogs by administering 200 mg/kg APAP, which resulted in a decrease in appetite and thirst, weakness, reduced activity, jaundice, abdominal swelling, and palpable ascites. Dogs were randomly divided into four groups (*n* = 3): a healthy canine NC group without drug and cell injection, an APAP group with acetaminophen injection, an APAP+CMV group with acetaminophen injection followed by MSC/CMV transplantation for control, and an ADSC co-expressing GPX3 and CD47 labeled APAP+GPX3&CD47 after acetaminophen injection. We then administered MSCs intravenously to the treatment group 14 days after the model was established, and samples were collected at 14 days. The results showed that the livers of the APAP-treated dogs were swollen and larger in size, with a softer texture, and more prone to rupture compared to the other groups (Figure 4a). The livers of the treatment groups showed different degrees of alleviation. The Organ Weight Index is a biological and medical index used to evaluate organ size relative to the body size. This index is particularly important in research, especially when organ growth or shrinkage is related to certain health conditions. The Organ Weight Index of the APAP group was significantly higher than that of the NC group, indicating that the liver was swollen and larger, whereas the Organ Weight Index of the GPX3&CD47 groups was significantly lower than that of the APAP group (Figure 4b). The ratio of AST to ALT reflects the degree of liver damage, and the results showed that the AST/ALT ratio significantly increased after APAP-induced liver injury, suggesting the presence of inflammation, damage, or dysfunction in the liver. However, transplantation of MSCs to different degrees significantly reduced the APAP-induced increase in AST/ALT caused by APAP (Figure 4c).

Histopathological examination can provide direct observation of the structural and morphological changes in the liver tissue under a microscope. HE staining results showed that in the APAP group, central lobular areas (near the central vein of the small leaf) in the liver exhibited predominant hepatocyte necrosis. Necrotic hepatocytes appeared as condensed or dissolved nuclei, and the necrotic areas were usually surrounded by the infiltration of inflammatory cells, accompanied by red blood cell infiltration and vascular rupture (Figure 4d). Co-expression of GPX3 and CD47 in MSCs effectively alleviated hepatocyte necrosis and degeneration, with reduced inflammatory and red blood cell infiltration, fewer vascular ruptures, and bleeding (Figure 4d).

Next, a DILI model was established in dogs (n = 5) by administering high-dose APAP (400 mg/kg) and MSCs as a treatment. For the treatment group, 1 × 10^7^ cells were intravenously injected into each dog daily and the survival status of the dogs was recorded (Figure 4e). Within 24 h of injection, the dogs in the APAP group showed symptoms of vomiting and coma, and 2 dogs died on the first day. All dogs in the APAP group died within 3 days, while 60% of the dogs in the CMV group survived, with one death on the 3rd and 5th days. The survival rate was 80% in the GPX3&CD47 groups, with one death on the 4th day. The results showed that GPX3 and CD47 co-expressing MSCs effectively reduced the number of dog deaths caused by high-dose intraperitoneal injection of APAP.

To further investigate the effects of MSC/GPX3 and CD47 transplantation on APAP-induced DILI, we established a mouse model and collected liver tissue samples seven days later. Liver tissues were embedded in paraffin and stained.

**Figure 5:**
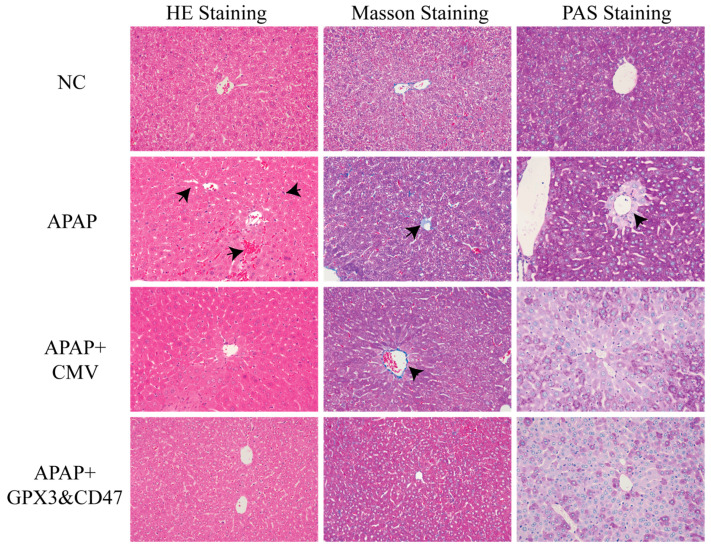
Histological examination of the mouse liver tissue (400× magnification), the arrow shows the lesion area.

The HE staining results showed the morphology of the liver tissue sections (Figure 5 left). In the NC group, the liver tissue structure was clear, and the liver cells were arranged in a regular pattern without obvious signs of necrosis or inflammatory cell infiltration. The central vein and hepatic sinusoids were normally arranged. In contrast, hepatocytes in the APAP group were significantly swollen with areas of necrosis, and inflammatory cell infiltration was observed around the central vein. There was also a large number of red blood cells in the hepatocytes, indicating that APAP-induced liver injury caused hepatocyte swelling, necrosis, bleeding, and inflammation. Compared to the APAP model group, the CMV group had fewer areas of liver necrosis and less inflammatory cell infiltration, and the arrangement of liver cells improved slightly, showing a certain protective effect. Compared to the APAP and CMV treatment groups, the MSC/GPX3&CD47 group showed a marked improvement in liver tissue structure, with a further reduction in areas of liver necrosis and inflammatory cell infiltration, and a more regular arrangement of hepatocytes, indicating that this treatment group had a more significant effect in reducing liver injury.

Masson staining was used to study the changes in liver fibrosis during the liver injury caused by APAP. It can be seen (Figure 5 middle) that there was almost no blue collagen fiber staining in the liver tissue except blood vessels in the NC group, indicating that there was no fibrosis in the normal liver tissue. In the liver injury caused by APAP, the staining of blue collagen fibers was increased by Masson staining, indicating the occurrence of liver fibrosis, which is one of the common pathological phenomena after liver injury. Compared with the APAP group, the staining of collagen fibers in the liver sections of the MSC group decreased.

This indicates that the degree of fibrosis was reduced, suggesting that MSC transplantation has an inhibitory effect on liver fibrosis. MSCs co-expressing GPX3 and CD47 significantly reduced mouse liver collagen fiber production, and blue collagen fiber staining was significantly reduced.

The main significance of PAS staining is to detect glycogen stores in hepatocytes and evaluate hepatocyte function. Purplish red glycogen deposition is widely distributed and uniform in normal liver tissues (Figure 5, right). In the APAP model group, glycogen deposition decreased radially around the central vein, which may be related to damage of hepatic lobule caused by APAP. However, in the MSC/CMV and MSC/GPX3 & CD47 groups, glycogen deposition was generally reduced, which was different from the APAP model group. This may be due to the fact that the transplantation of MSCs alters the metabolic rate of liver cells and accelerates the breakdown of glycogen to cope with the demand for cell repair. Although fibrosis and damage were reduced, glycogen synthesis and reserves were not fully restored.

**Figure 6:**
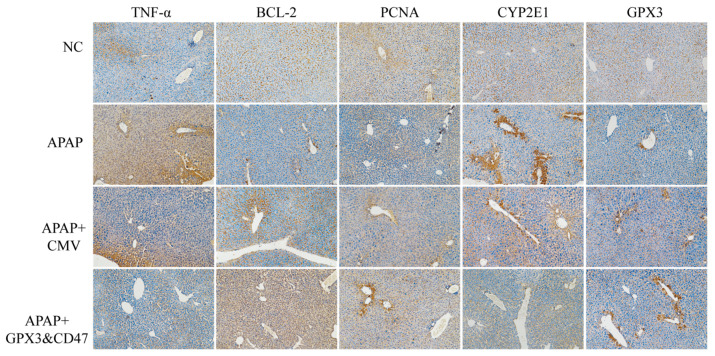
The expression levels of TNF-α, BCL-2, PCNA, CYP2E1, and GPX3 in liver tissue of mice were detected by immunohistochemistry (400× magnification).

The expression of TNF-α in the mouse liver was detected using immunohistochemistry. TNF-α (tumor necrosis factor-α) is a key pro-inflammatory cytokine that plays an important role in liver inflammation and injury. In DILI, an increase in TNF-α is usually closely related to the inflammatory response, apoptosis, and hepatocyte necrosis. TNF-α staining was very weak in the NC group, indicating that the expression of TNF-α in the normal control group was extremely low. In the APAP group, TNF-α staining showed significant brown DAB stained areas (Figure 6), showing that APAP induced a strong expression of TNF-α in the liver, suggesting a significant inflammatory response and liver injury. DAB staining in the CMV group was weakened, indicating that MSC/CMV transplantation reduced the inflammatory response in the liver. In the MSC/GPX3&CD47 group, the positive areas of TNF-α staining were further weakened, indicating that MSCs overexpressing GPX3 and CD47 had a stronger effect on inhibiting TNF-α expression and inflammatory response. This may indicate that these genetic modifications enhanced the anti-inflammatory effect of MSCs, which further alleviated liver injury by reducing TNF-α levels.

We then investigated the effects of MSC/GPX3&CD47 on mouse liver apoptosis. BCL-2 (B-cell lymphoma-2) is an anti-apoptotic protein that protects cells from apoptosis, mainly by inhibiting the permeability of the mitochondrial outer membrane, preventing the release of cytochrome c, and preventing the initiation of the apoptotic cascade. Here, we examined the expression of BCL-2 in liver tissue from different groups, and the results showed (Figure 6) that the positive signal was weak in the NC group, indicating that BCL-2 expression was lower in normal liver tissue. There was a slight increase in the BCL-2 positive signal in the APAP group; however, it was still weak. Compared to the APAP group, the BCL-2 staining signal was enhanced in the MSC treatment group, indicating that MSC transplantation effectively increased the expression level of BCL-2. The positive signal of BCL-2 was further enhanced in the MSC/GPX3&CD47 group, indicating that the gene-modified MSCs further increased the expression of BCL-2, which can be attributed to the overexpression of GPX3 and CD47, which not only enhanced the antioxidant capacity but also activated the anti-apoptotic mechanism. Furthermore, the liver was protected from APAP-induced damage caused by APAP.

To determine whether genetically modified MSCs could improve cell proliferation to promote the repair of damaged liver tissue, we detected the expression of PCNA. Proliferating cell nuclear antigen (PCNA) is an essential factor in DNA replication and repair. Most positive areas in the NC group were distributed uniformly around the central venous sinus (Figure 6), showing a relatively high level of expression. This situation was completely different in the APAP group. There were hardly any positive areas, indicating that the cell proliferation process was inhibited. Following the transplantation of MSCs, a significant shift was observed, with the expression of PCNA being remarkably elevated in the MSC/GPX3&CD47 group. These results indicate that co-expressing MSCs can ameliorate liver injury by promoting cell proliferation.

CYP2E1 (Cytochrome P450 2E1) is an important liver metabolic enzyme that is mainly responsible for the metabolism of APAP into the toxic metabolite N-acetyl-P-benzoquinone imine (NAPQI). NAPQI is the main cause of APAP causing liver injury and is mainly decomposed by glutathione (GSH) in the liver. The results showed (Figure 6) that in the normal liver, the expression of CYP2E1 was low, and there was almost no positive staining signal. CYP2E1 expression significantly increased after APAP injection. This finding is consistent with the role of CYP2E1 in APAP metabolism. The dark brown areas of CYP2E1 staining in the CMV group were slightly weaker than those in the APAP group, indicating that MSC transplantation may have a regulatory effect on the expression of CYP2E1. However, the positive areas of CYP2E1 staining in the MSC/GPX3&CD47 group were further weakened, indicating that MSCs overexpressing GPX3 and CD47 had a stronger inhibitory effect on CYP2E1 expression. This may be because the antioxidant effect of GPX3 reduces oxidative stress caused by APAP metabolism, thereby reducing the induced expression of CYP2E1, or CD47 reduces the inflammatory response in the liver through immunomodulatory effects.

GPX3 (glutathione peroxidase 3) is an important antioxidant enzyme that catalyzes the reduction of hydrogen peroxide and organic peroxides by glutathione, thereby protecting cells from oxidative stress damage. The results showed that GPX3 expression was lower in normal liver tissues without inflammation and oxidative stress (Figure 6). The expression of GPX3 in the APAP group was higher than that in the NC group, indicating that the expression level of GPX3 in the liver was increased to alleviate oxidative stress caused by APAP in the APAP-induced DILI model. The strongest positive signal was observed in the MSC/GPX3&CD47 group, indicating that the expression of GPX3 was significantly increased after transplantation of MSCs co-expressing GPX3 and CD47. MSC/GPX3&CD47 expressed higher levels of GPX3 and showed greater resistance to oxidative stress. GPX3 is a secreted protein that directly enhances antioxidant capacity of the liver.

**Figure 7:**
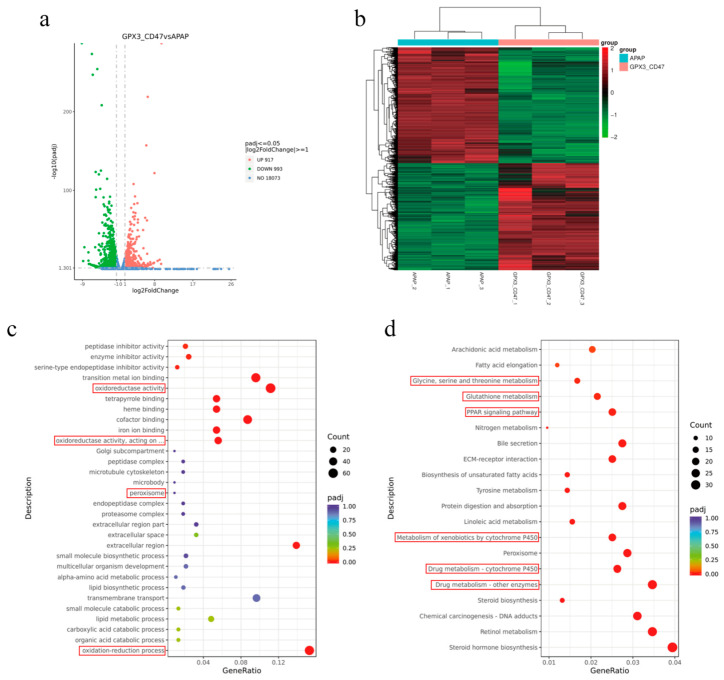
GPX3 and CD47 co-expression in MSCs alters hepatic transcriptional profiles in a DILI mouse model. (**a**) Differential gene volcano plot; (**b**) differential gene heatmap; (**c**) GO enrichment analysis; (**d**) KEGG enrichment analysis.

In this study, we performed transcriptome sequencing analysis of liver tissues in a mouse model of APAP-induced drug-induced liver injury (DILI) to comprehensively explore changes in gene expression and their biological significance. By comparing the gene expression profiles between the model group (APAP) and the treatment group (MSC/GPX3 &CD47), several significantly differentially expressed genes were identified, which played a role in several key biological pathways. A total of 1910 differentially expressed genes were identified by transcriptome sequencing, of which 917 were significantly upregulated and 993 were significantly downregulated (*p* < 0.05, log2FoldChange > 1) (Figure 7a). The gene heatmap showed significant between-group differences in samples with good sample reproducibility and low within-group differences (Figure 7b). GO analysis showed that peptidase inhibitor activity and transition metal ion binding were enriched for binding), peroxisome, oxidoreductase activity, and other terms. Among them, pathways related to REDOX reactions were significantly enriched (Figure 7c), such as oxidoreductase activity acting on paired donors. Oxidoreductase activity acting on paired donors is associated with the addition or reduction of oxygen molecules, peroxisomes, and oxidation-reduction processes. KEGG pathways were enriched in glycine, serine, and threonine metabolism, glutathione metabolism, PPAR signaling pathway, metabolism of xenobiotics by cytochrome P450, and drug metabolism-other enzymes (Figure 7d), which were mainly enriched in REDOX, inflammatory response, and drug metabolism.

Combined with GO and KEGG data, these differentially expressed genes were mainly enriched in glutathione (GSH) metabolism, oxidoreductase activity, inflammatory response, and drug metabolism enzymes. These results not only confirmed the therapeutic effect of MSC/GPX3 and CD47 on APAP-induced damaged hepatocytes observed in previous experiments but also confirmed the effect of MSC/GPX3 &CD47 on APAP-induced damaged hepatocytes. They also provide a comprehensive explanation of the underlying molecular mechanisms. After transplantation of MSC/GPX3 and CD47, the expression of key genes related to the regulation of the glutathione metabolic pathway and oxidoreductase activity in hepatocytes was significantly upregulated, which enhanced the anti-oxidative stress ability of hepatocytes and may help alleviate oxidative damage induced by APAP. In addition, the genes related to inflammatory response and drug metabolism enzyme activities were significantly downregulated, which was consistent with previous results, indicating that MSC/GPX3&CD47 transplantation alleviated liver inflammation and reduced the activities of drug metabolism enzymes, thus reducing further liver damage caused by drug metabolism poisons. In conclusion, MSC/GPX3&CD47 transplantation significantly enhanced the antioxidant capacity of hepatocytes and alleviated liver injury by regulating key genes such as glutathione metabolism, oxidoreductase activity, inflammatory response, and drug metabolism enzyme activity.

**Figure 8:**
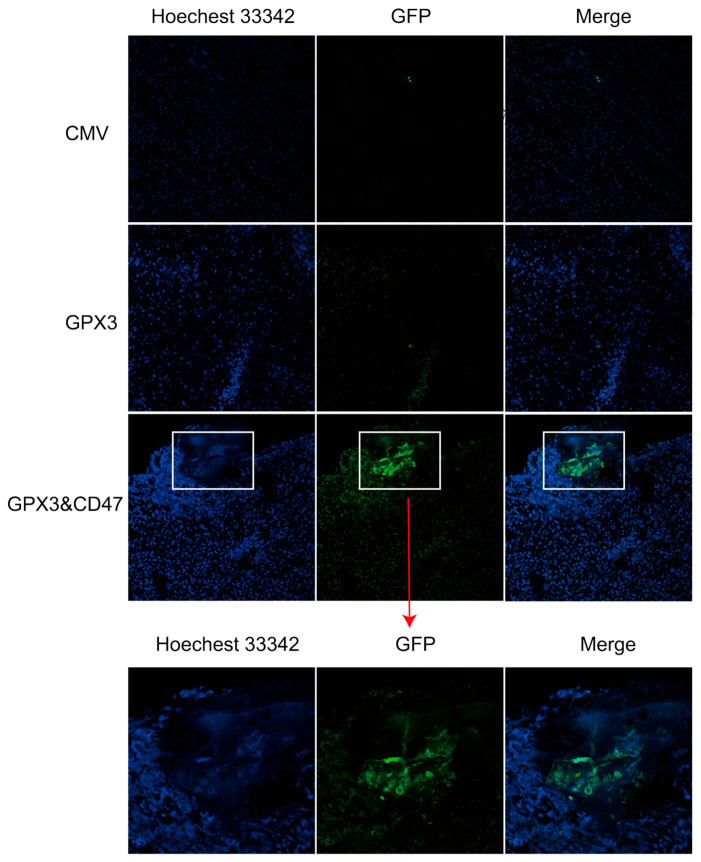
Assessment of in vivo survival of MSCs (200× magnification).

Finally, we previously demonstrated that overexpression of CD47 enhanced the ability of MSCs to evade macrophages in vitro, and we wanted to explore whether overexpression of CD47 could enhance the immune evasion ability of MSCs in vivo. In this study, MSCs with GFP green fluorescent protein were injected intravenously into mice at a dose of 1 × 10^6^ cells/mouse, and the livers were collected on day 14 for frozen section to observe the survival of MSCs in the liver. It can be seen that MSCs carrying PCDH-CMV empty vector as well as MSCs overexpressing the GPX3 gene survived less in the liver and most of the cells were eliminated. The green fluorescence signal was significantly enhanced in the GPX3&CD47 group, with more MSCs surviving, and these clusters of surviving MSCs were distributed in clumps rather than widely. The extensive aggregation of cells in the GPX3&CD47 group indicated that CD47 overexpression significantly enhanced the in vivo viability of these cells (Figure 8).

## 4. Discussion

Mesenchymal stem cells (MSCs) have demonstrated promising therapeutic effects and potential applications in the treatment of various diseases, particularly in addressing osteoarthritis [62], cardiovascular diseases [63,64], and diabetes, exhibiting robust capabilities in promoting the regeneration of damaged tissues. However, previous research has highlighted several challenges associated with the clinical use of MSCs, including cellular heterogeneity and immune rejection. Heterogeneity in MSCs refers to the instability in therapeutic outcomes due to variations in cell sources, donor health status, cell passages, and in vitro culture conditions [42]. Notably, continuous in vitro passaging of cells leads to a decline in proliferative capacity and other phenotypic changes, significantly impeding the efficacy of MSCs in vivo. Furthermore, while MSCs are generally considered to have low immunogenicity and typically do not elicit severe immune rejection, they are not entirely unrecognized by the host immune system. Studies have shown that MSCs do not persist after intravenous infusion, with the majority of systemically infused cells being cleared within 48 h [65,66] and can elicit immune memory, potentially resulting in the failure of subsequent MSC transplantations [67,68].

In this study, we overexpressed the GPX3 and CD47 genes in MSCs. Transcriptome analysis revealed that, compared to unmodified MSCs, overexpression of GPX3 and CD47 genes promoted the expression of genes related to the mitotic spindle, DNA replication, and cell cycle pathways. Simultaneously, upregulation was observed in pathways such as the p53 signaling pathway and glutathione metabolism, along with downregulation of apoptosis-related genes. These findings suggest that genetic modification with GPX3 and CD47 enhances DNA replication, cell proliferation, and antioxidant capabilities in MSCs, while inhibiting the expression of apoptosis-related genes. Notably, the upregulation of genes associated with the DNA replication pathway indicates that genetic modification significantly boosts the proliferative capacity of MSCs, contributing to their viability and adaptability during in vitro culture [69,70]. The significant downregulation of apoptosis pathway genes in gene-modified MSCs demonstrates their enhanced anti-apoptotic properties, which are crucial for improving cell survival rates and maintaining functionality [71]. It is worth noting that although the transcriptome level showed that the proliferation and antioxidant damage related genes were upregulated in GPX3 and CD47 overexpressing MSCs, further evidence to support cell proliferation and antioxidant damage is still lacking in our study. We will supplement the relevant evidence in the next study. In coculture experiments with RAW 264.7 cells, MSCs co-expressing GPX3 and CD47 exhibited significantly higher survival rates compared to unmodified MSCs and MSCs overexpressing only GPX3, indicating that overexpression of CD47 enhances the resistance of MSCs to macrophage phagocytosis.

Then, we conducted cell transplantation therapy for acetaminophen (APAP)-induced drug-induced liver injury (DILI) in mice and dogs. MSCs co-expressing GPX3 and CD47 effectively alleviated APAP-induced liver damage in dogs, significantly reducing mortality rates in dogs subjected to high-dose APAP modeling. Subsequently, we established a DILI model in mice, and histological results showed that MSC/GPX3&CD47 reduced inflammatory cell infiltration and fibrosis in liver tissue. Immunofluorescence analysis indicated that MSC/GPX3&CD47 resisted APAP-induced liver injury by inhibiting inflammation, modulating the activity of liver drug-metabolizing enzymes, and reducing cellular apoptosis. Furthermore, liver transcriptome analysis revealed that MSC/GPX3&CD47 upregulated genes related to glutathione (GSH) metabolism and catalase while downregulating genes associated with oxidative damage, inflammatory responses, and drug-metabolizing enzymes, thereby mitigating APAP-induced liver damage, which corroborated our previous experimental results. Lastly, compared to the in vivo survival rates of MSCs without CD47 overexpression, MSCs co-expressing GPX3 and CD47 in this study exhibited higher survival rates. This finding is consistent with the research by Chao et al., who observed a similar CD47-mediated immune evasion mechanism in hematopoietic stem cells (HSCs) [72].

It is noteworthy that, although our study demonstrates that overexpressing these two exogenous genes can enhance the proliferation, anti-apoptotic properties, and immune evasion capabilities of MSCs, it cannot avoid recognition and clearance by other immune cells in the host. For instance, cultured and expanded MSCs express low levels of MHC class I and are negative for MHC class II, but when exposed to interferon-gamma (IFN-γ) or tumor necrosis factor-alpha (TNF-α), MSCs significantly upregulate their expression of both MHC class I and class II, making them susceptible to recognition and clearance by T-cell receptors [73,74]. This study found that MSCs colonizing the liver alleviate drug-induced liver injury through multiple pathways, yet the fate of MSCs in vivo remains unclear. Furthermore, research on the use of MSCs in veterinary medicine requires tracking their long-term efficacy and side effects to better evaluate their therapeutic efficacy and safety.

## 5. Conclusions

In summary, we established MSCs co-expressing GPX3 and CD47. Compared with MSCs without GPX3 and CD47 gene modification, MSC/GPX3&CD47 exhibited enhanced proliferation, anti-apoptotic properties relevant gene expression, and improved immune evasion capabilities. In the treatment of drug-induced liver injury models in mice and dogs, MSC/GPX3&CD47 significantly reduced liver inflammation and apoptosis levels, enhanced liver antioxidant capacity, and improved the survival efficiency of MSCs in vivo, thereby alleviating APAP-induced drug-induced liver injury. In short, we have established a novel MSC cell strain through gene editing, which has broad research and application prospects for the treatment of drug-induced liver injury in the field of veterinary medicine.

## Data Availability

The datasets presented in this article are available from the Wiley Online Library or the author.

## Supplementary Materials

The following supporting information can be downloaded at: https://www.mdpi.com/article/10.3390/vetsci12020149/s1, File S1: Supporting Information.

## References

[1] Devarbhavi H., Asrani S.K., Arab J.P., Nartey Y.A., Pose E., Kamath P.S. (2023). Global burden of liver disease: 2023 update. J. Hepatol..

[2] Asrani S.K., Devarbhavi H., Eaton J., Kamath P.S. (2019). Burden of liver diseases in the world. J. Hepatol..

[3] Hoofnagle J.H., Björnsson E.S. (2019). Drug-induced liver injury—Types and phenotypes. N. Engl. J. Med..

[4] Björnsson H.K., Björnsson E.S. (2022). Drug-induced liver injury: Pathogenesis, epidemiology, clinical features, and practical management. Eur. J. Intern. Med..

[5] Li X., Tang J., Mao Y. (2022). Incidence and risk factors of drug-induced liver injury. Liver Int..

[6] Ekor M. (2014). The growing use of herbal medicines: Issues relating to adverse reactions and challenges in monitoring safety. Front. Pharmacol..

[7] Norman B.H. (2020). Drug induced liver injury (DILI). Mechanisms and medicinal chemistry avoidance/mitigation strategies. J. Med. Chem..

[8] Dahlin D.C., Miwa G.T., Lu A.Y., Nelson S.D. (1984). N-acetyl-p-benzoquinone imine: A cytochrome p-450-mediated oxidation product of acetaminophen. Proc. Natl. Acad. Sci. USA.

[9] Björnsson E.S. (2015). Drug-induced liver injury: An overview over the most critical compounds. Arch. Toxicol..

[10] Chiew A.L., Gluud C., Brok J., Buckley N.A. (2018). Interventions for paracetamol (acetaminophen) overdose. Cochrane Database Syst. Rev..

[11] Larson A.M., Polson J., Fontana R.J., Davern T.J., Lalani E., Hynan L.S., Reisch J.S., Schiødt F.V., Ostapowicz G., Shakil A.O. (2005). Acetaminophen-induced acute liver failure: Results of a united states multicenter, prospective study. Hepatology.

[12] Dart R.C., Erdman A.R., Olson K.R., Christianson G., Manoguerra A.S., Chyka P.A., Caravati E.M., Wax P.M., Keyes D.C., Woolf A.D. (2006). Acetaminophen poisoning: An evidence-based consensus guideline for out-of-hospital management. Clin. Toxicol..

[13] Vincenzi B., Russo A., Terenzio A., Galvano A., Santini D., Vorini F., Antonelli-Incalzi R., Vespasiani-Gentilucci U., Tonini G. (2018). The use of SAMe in chemotherapy-induced liver injury. Crit. Rev. Oncol. Hematol..

[14] Björnsson H.K., Gudbjornsson B., Björnsson E.S. (2022). Infliximab-induced liver injury: Clinical phenotypes, autoimmunity and the role of corticosteroid treatment. J. Hepatol..

[15] Hu P.F., Xie W.F. (2019). Corticosteroid therapy in drug-induced liver injury: Pros and cons. J. Dig. Dis..

[16] Karkhanis J., Verna E.C., Chang M.S., Stravitz R.T., Schilsky M., Lee W.M., Brown R.S.J. (2014). Steroid use in acute liver failure. Hepatology.

[17] Brahmer J.R., Lacchetti C., Schneider B.J., Atkins M.B., Brassil K.J., Caterino J.M., Chau I., Ernstoff M.S., Gardner J.M., Ginex P. (2018). Management of immune-related adverse events in patients treated with immune checkpoint inhibitor therapy: American society of clinical oncology clinical practice guideline. J. Clin. Oncol..

[18] van Gerven N.M.F., Verwer B.J., Witte B.I., van Hoek B., Coenraad M.J., van Erpecum K.J., Beuers U., van Buuren H.R., de Man R.A., Drenth J.P.H. (2013). Relapse is almost universal after withdrawal of immunosuppressive medication in patients with autoimmune hepatitis in remission. J. Hepatol..

[19] Gentile P. (2021). New strategies in plastic surgery: Autologous adipose-derived mesenchymal stem cells contained in fat grafting improves symptomatic scars. Front. Biosci.-Landmark.

[20] Gentile P. (2021). Breast silicone gel implants versus autologous fat grafting: Biomaterials and bioactive materials in comparison. J. Clin. Med..

[21] Lee W.S., Kim H.J., Kim K.I., Kim G.B., Jin W. (2019). Intra-articular injection of autologous adipose tissue-derived mesenchymal stem cells for the treatment of knee osteoarthritis: A phase IIb, randomized, placebo-controlled clinical trial. Stem Cells Transl. Med..

[22] Zhou C., Zhang B., Yang Y., Jiang Q., Li T., Gong J., Tang H., Zhang Q. (2023). Stem cell-derived exosomes: Emerging therapeutic opportunities for wound healing. Stem Cell Res. Ther..

[23] Xu X., Li X., Wang J., He X., Sun H., Chen F. (2019). Concise review: Periodontal tissue regeneration using stem cells: Strategies and translational considerations. Stem Cells Transl. Med..

[24] Spees J.L., Lee R.H., Gregory C.A. (2016). Mechanisms of mesenchymal stem/stromal cell function. Stem Cell Res. Ther..

[25] Xing L., Cui R., Peng L., Ma J., Chen X., Xie R., Li B. (2014). Mesenchymal stem cells, not conditioned medium, contribute to kidney repair after ischemia-reperfusion injury. Stem Cell Res. Ther..

[26] Sacchetti B., Funari A., Michienzi S., Di Cesare S., Piersanti S., Saggio I., Tagliafico E., Ferrari S., Robey P.G., Riminucci M. (2007). Self-renewing osteoprogenitors in bone marrow sinusoids can organize a hematopoietic microenvironment. Cell.

[27] Daley G.Q. (2015). Stem cells and the evolving notion of cellular identity. Philos. Trans. R. Soc. B Biol. Sci..

[28] Crisan M., Yap S., Casteilla L., Chen C., Corselli M., Park T.S., Andriolo G., Sun B., Zheng B., Zhang L. (2008). A perivascular origin for mesenchymal stem cells in multiple human organs. Cell Stem Cell.

[29] Kobolak J., Dinnyes A., Memic A., Khademhosseini A., Mobasheri A. (2016). Mesenchymal stem cells: Identification, phenotypic characterization, biological properties and potential for regenerative medicine through biomaterial micro-engineering of their niche. Methods.

[30] Liu J., Gao J., Liang Z., Gao C., Niu Q., Wu F., Zhang L. (2022). Mesenchymal stem cells and their microenvironment. Stem Cell Res. Ther..

[31] Tao X., Li W., Su J., Jin C., Wang X., Li J., Hu J., Xiang Z., Lau J.T.Y., Hu Y. (2009). Clonal mesenchymal stem cells derived from human bone marrow can differentiate into hepatocyte-like cells in injured livers of SCID mice. J. Cell. Biochem..

[32] Aurich H., Sgodda M., Kaltwasser P., Vetter M., Weise A., Liehr T., Brulport M., Hengstler J.G., Dollinger M.M., Fleig W.E. (2009). Hepatocyte differentiation of mesenchymal stem cells from human adipose tissue in vitro promotes hepatic integration in vivo. Gut.

[33] Ichikawa A., Neo S., Nukui R., Yamada Y., Nitta S., Iwaki H., Yanagi Y., Nakayama K., Sato S., Tateishi S. (2022). Establishment of large canine hepatocyte spheroids by mixing vascular endothelial cells and canine adipose-derived mesenchymal stem cells. Regen. Ther..

[34] Zhou R., Li Z., He C., Li R., Xia H., Li C., Xiao J., Chen Z. (2014). Human umbilical cord mesenchymal stem cells and derived hepatocyte-like cells exhibit similar therapeutic effects on an acute liver failure mouse model. PLoS ONE.

[35] Afshari A., Shamdani S., Uzan G., Naserian S., Azarpira N. (2020). Different approaches for transformation of mesenchymal stem cells into hepatocyte-like cells. Stem Cell Res. Ther..

[36] Aurich I., Mueller L.P., Aurich H., Luetzkendorf J., Tisljar K., Dollinger M.M., Schormann W., Walldorf J., Hengstler J.G., Fleig W.E. (2007). Functional integration of hepatocytes derived from human mesenchymal stem cells into mouse livers. Gut.

[37] Wang L., Li Y., Xu M., Deng Z., Zhao Y., Yang M., Liu Y., Yuan R., Sun Y., Zhang H. (2021). Regulation of inflammatory cytokine storms by mesenchymal stem cells. Front. Immunol..

[38] Caplan A.I., Dennis J.E. (2006). Mesenchymal stem cells as trophic mediators. J. Cell. Biochem..

[39] Banfi A., Bianchi G., Galotto M., Cancedda R., Quarto R. (2001). Bone marrow stromal damage after chemo/radiotherapy: Occurrence, consequences and possibilities of treatment. Leuk. Lymphoma.

[40] Zhou T., Yuan Z., Weng J., Pei D., Du X., He C., Lai P. (2021). Challenges and advances in clinical applications of mesenchymal stromal cells. J. Hematol. Oncol..

[41] Wagner W., Horn P., Castoldi M., Diehlmann A., Bork S., Saffrich R., Benes V., Blake J., Pfister S., Eckstein V. (2008). Replicative senescence of mesenchymal stem cells: A continuous and organized process. PLoS ONE.

[42] Li J., Wu Z., Zhao L., Liu Y., Su Y., Gong X., Liu F., Zhang L. (2023). The heterogeneity of mesenchymal stem cells: An important issue to be addressed in cell therapy. Stem Cell Res. Ther..

[43] Oh J.Y., Kim H., Lee H.J., Lee K., Barreda H., Kim H.J., Shin E., Bae E., Kaur G., Zhang Y. (2022). MHC class i enables MSCs to evade NK-cell–mediated cytotoxicity and exert immunosuppressive activity. Stem Cells.

[44] Reddy A.T., Lakshmi S.P., Banno A., Reddy R.C. (2018). Role of GPx3 in PPARγ-induced protection against COPD-associated oxidative stress. Free Radic. Bio. Med..

[45] Qi X., Ng K.T., Lian Q., Li C.X., Geng W., Ling C.C., Yeung W.H., Ma Y.Y., Liu X.B., Liu H. (2018). Glutathione peroxidase 3 delivered by hiPSC-MSCs ameliorated hepatic IR injury via inhibition of hepatic senescence. Theranostics.

[46] Leung C.S., Li J., Xu F., Wong A., Lui K.O. (2019). Ectopic expression of recipient CD47 inhibits mouse macrophage-mediated immune rejection against human stem cell transplants. FASEB J..

[47] Chao M.P., Alizadeh A.A., Tang C., Myklebust J.H., Varghese B., Gill S., Jan M., Cha A.C., Chan C.K., Tan B.T. (2010). Anti-CD47 antibody synergizes with rituximab to promote phagocytosis and eradicate non-hodgkin lymphoma. Cell.

[48] Beckett A.N., Chockley P., Pruett-Miller S.M., Nguyen P., Vogel P., Sheppard H., Krenciute G., Gottschalk S., Derenzo C. (2023). CD47 expression is critical for CAR t-cell survival in vivo. J. Immunother. Cancer.

[49] Komori S., Saito Y., Nishimura T., Respatika D., Endoh H., Yoshida H., Sugihara R., Iida-Norita R., Afroj T., Takai T. (2023). CD47 promotes peripheral t cell survival by preventing dendritic cell-mediated t cell necroptosis. Proc. Natl. Acad. Sci. USA.

[50] Wei Y., Fang J., Cai S., Lv C., Zhang S., Hua J. (2016). Primordial germ cell-like cells derived from canine adipose mesenchymal stem cells. Cell Prolif..

[51] Kou Z., Li B., Aierken A., Tan N., Li C., Han M., Jing Y., Li N., Zhang S., Peng S. (2023). Mesenchymal stem cells pretreated with collagen promote skin wound-healing. Int. J. Mol. Sci..

[52] Li C., Li B., Han M., Tian H., Gao J., Han D., Ling Z., Jing Y., Li N., Hua J. (2024). SPARC overexpression in allogeneic adipose-derived mesenchymal stem cells in dog dry eye model induced by benzalkonium chloride. Stem Cell Res. Ther..

[53] Fang J., Yan Y., Teng X., Wen X., Li N., Peng S., Liu W., Donadeu F.X., Zhao S., Hua J. (2018). Melatonin prevents senescence of canine adipose-derived mesenchymal stem cells through activating NRF2 and inhibiting ER stress. Aging.

[54] Yu Y., Wang D., Li H., Liu Y., Xiang Z., Wu J., Jing X. (2018). IPSC-MSC inhibition assessment in raw 264.7 cells following oxygen and glucose deprivation reveals a distinct function for cardiopulmonary resuscitation. Mol. Med. Rep..

[55] Urrunaga N.H., Jadeja R.N., Rachakonda V., Ahmad D., Mclean L.P., Cheng K., Shah V., Twaddell W.S., Raufman J., Khurana S. (2015). M1 muscarinic receptors modify oxidative stress response to acetaminophen-induced acute liver injury. Free Radic. Bio. Med..

[56] Satué M., Schüler C., Ginner N., Erben R.G. (2019). Intra-articularly injected mesenchymal stem cells promote cartilage regeneration, but do not permanently engraft in distant organs. Sci. Rep..

[57] Kim S.K., Pak H., Park J.H., Fang Y.F., Kim G.I., Park Y.D., Hwang C., Kim Y., Kim B.S. (2010). Cardiac cell therapy with mesenchymal stem cell induces cardiac nerve sprouting, angiogenesis, and reduced connexin43-positive gap junctions, but concomitant electrical pacing increases connexin43-positive gap junctions in canine heart. Cardiol. Young.

[58] Jung D., Ha J., Kang B., Kim J., Quan F., Lee J., Woo E., Park H. (2009). A comparison of autologous and allogenic bone marrow-derived mesenchymal stem cell transplantation in canine spinal cord injury. J. Neurol. Sci..

[59] Lee S.H., Kim Y., Rhew D., Kim A., Jo K.R., Yoon Y., Choi K.U., Jung T., Kim W.H., Kweon O. (2016). Impact of local injection of brain-derived neurotrophic factor-expressing mesenchymal stromal cells (MSCs) combined with intravenous MSC delivery in a canine model of chronic spinal cord injury. Cytotherapy.

[60] Liu C., Zhang X., Tan Q., Xu W., Zhou C., Luo M., Li X., Huang R., Zeng X. (2017). NF-κb pathways are involved in m1 polarization of RAW 264.7 macrophage by polyporus polysaccharide in the tumor microenvironment. PLoS ONE.

[61] Raschke W.C., Baird S., Ralph P., Nakoinz I. (1978). Functional macrophage cell lines transformed by abelson leukemia virus. Cell.

[62] Toh W.S., Lee E.H., Guo X.M., Chan J.K., Yeow C.H., Choo A.B., Cao T. (2010). Cartilage repair using hyaluronan hydrogel-encapsulated human embryonic stem cell-derived chondrogenic cells. Biomaterials.

[63] Li Q., Hou H., Li M., Yu X., Zuo H., Gao J., Zhang M., Li Z., Guo Z. (2021). CD73(+) mesenchymal stem cells ameliorate myocardial infarction by promoting angiogenesis. Front. Cell Dev. Biol..

[64] Penn M.S., Ellis S., Gandhi S., Greenbaum A., Hodes Z., Mendelsohn F.O., Strasser D., Ting A.E., Sherman W. (2012). Adventitial delivery of an allogeneic bone marrow-derived adherent stem cell in acute myocardial infarction: Phase i clinical study. Circ. Res..

[65] Toma C., Wagner W.R., Bowry S., Schwartz A., Villanueva F. (2009). Fate of culture-expanded mesenchymal stem cells in the microvasculature: In vivo observations of cell kinetics. Circ. Res..

[66] Lee R.H., Pulin A.A., Seo M.J., Kota D.J., Ylostalo J., Larson B.L., Semprun-Prieto L., Delafontaine P., Prockop D.J. (2009). Intravenous hMSCs improve myocardial infarction in mice because cells embolized in lung are activated to secrete the anti-inflammatory protein TSG-6. Cell Stem Cell.

[67] Zangi L., Margalit R., Reich-Zeliger S., Bachar-Lustig E., Beilhack A., Negrin R., Reisner Y. (2009). Direct imaging of immune rejection and memory induction by allogeneic mesenchymal stromal cells. Stem Cells.

[68] Nauta A.J., Westerhuis G., Kruisselbrink A.B., Lurvink E.G., Willemze R., Fibbe W.E. (2006). Donor-derived mesenchymal stem cells are immunogenic in an allogeneic host and stimulate donor graft rejection in a nonmyeloablative setting. Blood.

[69] Schallmoser K., Bartmann C., Rohde E., Bork S., Guelly C., Obenauf A.C., Reinisch A., Horn P., Ho A.D., Strunk D. (2010). Replicative senescence-associated gene expression changes in mesenchymal stromal cells are similar under different culture conditions. Haematologica.

[70] Shilina M.A., Grinchuk T.M., Anatskaya O.V., Vinogradov A.E., Alekseenko L.L., Elmuratov A.U., Nikolsky N.N. (2018). Cytogenetic and transcriptomic analysis of human endometrial MSC retaining proliferative activity after sublethal heat shock. Cells.

[71] Sepúlveda J.C., Tomé M., Fernández M.E., Delgado M., Campisi J., Bernad A., González M.A. (2014). Cell senescence abrogates the therapeutic potential of human mesenchymal stem cells in the lethal endotoxemia model. Stem Cells.

[72] Chao M.P., Alizadeh A.A., Tang C., Jan M., Weissman-Tsukamoto R., Zhao F., Park C.Y., Weissman I.L., Majeti R. (2011). Therapeutic antibody targeting of CD47 eliminates human acute lymphoblastic leukemia. Cancer Res..

[73] Le Blanc K., Tammik C., Rosendahl K., Zetterberg E., Ringdén O. (2003). HLA expression and immunologic properties of differentiated and undifferentiated mesenchymal stem cells. Exp. Hematol..

[74] Chan J.L., Tang K.C., Patel A.P., Bonilla L.M., Pierobon N., Ponzio N.M., Rameshwar P. (2006). Antigen-presenting property of mesenchymal stem cells occurs during a narrow window at low levels of interferon-gamma. Blood.

