# Supplementary material for: Mesenchymal Stem Cells with Simultaneous Overexpression of GPX3 and CD47 for the Treatment of Drug-Induced Acute Liver Injury
Source: Vet Sci. 2025 Feb 10;12(2):149. doi: 10.3390/vetsci12020149 (PMC11861084; doi:10.3390/vetsci12020149)
Supplement: Supplementary file 1 [file vetsci-12-00149-s001.zip › Supporting Information.pdf]

Supporting Information

Mesenchymal Stem Cells with Simultaneous Overexpression of GPX3 and CD47 for the Treatment of Drug-Induced Acute Liver Injury

Yuanxiang Jing [1], Balun Li [1], Aili Aierken [1,2], Zengyu Zhang [1], Dongyao Han [1], Zixi Lin [1], Jiaqi Gao [1], Hongkai Tian [1] and Jinlian Hua [1,*]

1 College of Veterinary Medicine, Shanxi Centre of Stem Cells Engineering & Technology, Northwest A&F University, Yangling 712100, China
2 Key Laboratory of Pathogenesis, Prevention and Treatment of High Incidence Diseases in Central Asia, Xinjiang Medical University, Urumqi 830000, China
* Correspondence: jinlianhua@nwsuaf.edu.cn

1. Material and Method

1.1 Materials

Reagents: fetal bovine serum, L-glutamine, non-essential amino acids, BSA, DMEM high glucose basal medium, alph-MEM medium (Gibco Company, USA); Agarose; DNA Ladder Marker Color, Mixed Protein marker 11-180 KD, (Beijing Xolaibo Technology Co., LTD.); Super PCR Mix; Ethidium bromide (Shenzhen Jianzhu Technology Co., LTD.); Hydrochloric acid, glacial acetic acid; Acetaminophen, sodium chloride, potassium chloride (Shanghai Yuanye Biotechnology Co., LTD.); Alanine aminotransferase, aspartate aminotransferase, catalase, glutathione, glutathione peroxidase, superoxide dismutase (Nanjing Jiancheng Bioengineering Institute); Hematoxylin and eosin (Nanjing Jinyibai Biotechnology Co., LTD.);

Animals: The experimental animals included Kunming (KM) male mice (25±2 g), which were provided by Chengdu Dashuo Experimental Animal Co., LTD. And Chinese rural dog (3.5±1 kg), which was purchased from the dog market. Before the start of the experiment, all animals underwent adaptive feeding for 1 week and underwent a complete physical examination to ensure that their health status before the experiment was normal and to rule out any health problems that could affect the reliability of the experiment. To ensure the fairness of the results, animals were completely randomized. During the

adaptive feeding period, all experimental animals were kept in clean conventional animal facilities with free access to water and quantitative feeding conditions.

Kit: Nuclear staining Hoechst33342 (Beijing Solaibao Technology Co., LTD., Beijing); Reverse transcription kit (Tiangen Biochemical Technology Co., LTD., Beijing); RNA extraction using Trizol, real-time quantitative PCR kit (Takata Company, Japan); RIPA lysate (Biyuntian Co., China).

HRP labeled sheep anti-rabbit/mouse secondary antibody (Boorsen, China) was used for detection. Alexa Fluor® 594-labeled goat anti-rabbit IgG (Zhongshan Jinqiao, China); Alexa Fluor® 594-labeled goat anti-mouse IgG (Zhongshan Jinqiao, China); IL6 (Wuhan Sanying Company); GPX3 (Wuhan Sanying Company); CYP2E1 (Santacurz, USA); TNFα (Immunoway, USA); BCL2 (Immunoway, United States); CASPASE3 (Boorson, China).

1.2 Establishment of MSC/GPX3&CD47

Canine lung and kidney tissues were rapidly frozen in liquid nitrogen, and the tissue was ground to a fine powder using a mortar and pestle in the frozen state. The ground tissue was immediately transferred to a precooled 1.5 mL sterile centrifuge tube. Add 1 mL Trizol reagent (1 mL Trizol per 50-100 mg tissue) to the centrifuge tube and mix gently to ensure that the tissue is completely immersed in Trizol. Centrifuge tubes containing Trizol and tissue were left at room temperature for 5 min to ensure adequate lysis of the cells and homogenization of the samples. 0.2 mL of chloroform was added to each 1 mL of Trizol. Close the cap of the centrifuge tube and shake thoroughly for 15 seconds. It was allowed to stand at room temperature for 2-3 min to facilitate phase separation. Centrifugation was performed at 12,000 rpm (approximately 13,400 × g) for 15 min at 4 ℃. After centrifugation, the samples were divided into three layers: an upper colorless aqueous phase, an intermediate white protein phase, and a lower red organic phase. The upper aqueous phase (approximately 500 to 600 μL) was carefully removed and transferred to a new sterile 1.5 mL centrifuge tube, taking care to avoid touching the protein phase. An equal volume of isopropanol (about 500 to 600 μL) was added to the aqueous phase, gently mixed, and left at room temperature for 10 min to precipitate the RNA. Centrifugation was performed at 12,000 rpm for 10 min at 4 ℃ At this point the RNA will form a precipitate, located at the bottom of the tube. The supernatant was carefully decanted, taking care not to perturb the RNA precipitation. 1 mL of 75% ethanol was added to the RNA precipitate and gently mixed. Centrifugation was performed at 7500 rpm for 5 min at 4 ℃, followed by careful decantation of the supernatant, inversion of the tube on a paper

towel, and air drying of the RNA precipitate for 5-10 min, taking care not to allow the precipitate to dry completely so as not to affect dissolution. An appropriate amount of DEPC-treated sterile water (20 to 50 μL) was added to the RNA precipitate, and the mixture was gently blown to ensure complete dissolution of the RNA. The dissolved RNA was stored at − 80 ℃until further use.

The RNA treated above was extracted according to the instructions of the Tianyen cDNA synthesis kit and stored at -20℃. The GPX3 gene (NM_001164454.1) and CD47 gene (NM_001080721.1) were searched in NCBI database, and the coding region of the GPX3 gene was used as a template to design primers for PCR amplification. The gene amplification primers are GPX3-F: AAAACAACTACGGTTTATGTGAAGGG, GPX3-R: TACTGACTGATTATTCATGCCCT, with an amplification length of 681 bp; CD47-F: GGAACTGCTGTTGGCGGT, CD47-R: AGCAACTCCAACTCCCAGT, with an amplification length of 912 bp.

PCDH-CMV-EF1-Puro and PCDH-CMV-EF1-NEO vectors were used to construct PCDH-CMV-GPX3-EF1-Puro and PCDH-CMV-CD47-EF1-NEO lentiviral vectors, respectively. EcoRI and XbaI restriction enzymes were used to cleave the vector PCDH-CMV-EF1-Puro, and XbaI and BamHI restriction enzymes were used to cleave the vector PCDH-CMV-EF1-NEO. After the reaction, the system was subjected to nucleic acid electrophoresis, and the DNA fragments were recovered by gel at the end of electrophoresis. The recovered linearized vector was connected with the cloned gene fragment by homologous recombination according to the manufacturer's instructions.

The identified GPX3 target plasmid was mixed with PAX2, VSVG, 20 μl PEI and 500 μl Opti-MEM in a 1.5 ml centrifuge tube for 20 minutes. The mixture was added to 293T cells cultured in logarithmic growth phase at 37 ℃ and 5% CO2 for 12 hours, and then changed to DMEM medium for further culture for 48-72 hours. The supernatant containing lentivirus particles was mixed with α-MEM at a ratio of 1:1 and then added to MSCs cultured to logarithmic growth phase. After 3 days of continuous culture, the positive cells were screened by puromycin. The CD47 target plasmid was transfected into GPX3 over-expressing MSCs screened as positive as described above, and positive cells were selected using G418.

1.3 In vitro co-culture experiments

Firstly, The MSC/GPX3 and MSC/GPX3&CD47 cells cultured to logarithmic growth phase were stained with PKH 26. The procedure was as follows: the cells were digested with trypsin, the trypsin was

discarded after centrifugation, and the cell suspension was resuspended in α-MEM medium. The PKH 26 dye was diluted with dilution C in a certain ratio, usually 1:250 to 1:500, according to the kit instructions. The mixture was homogeneous to avoid bubble formation, and 5 to 10 times the volume of α-MEM was added immediately after the end of the staining time to terminate the staining reaction. Mix well. Excess staining solution was removed by centrifugation, and the cells were re-suspended to a new culture dish for culture. RAW 264.7 cells were added to the stained MSCs at a ratio of 1:1 and cultured together. The medium was changed daily and the cell status was observed under fluorescence.

1.4 In vivo canine and mouse DILI model

After adaptive feeding, all the mice and dogs were randomly divided into :① normal control (NC) group; ②APAP group; ③MSCs treatment group; ④MSC/GPX3&CD47 combined treatment group; Eight mice or three dogs were used in each group. Except for the NC group, the other three groups were fasted for 12 hours and then intraperitoneally injected with 200mg/kg APAP to establish the model. The cells were injected at 24 hours after modeling, and the amount of transplanted cells was 1×10^6 cells/mouse through the tail vein, and 1×10^7 cells/dog was transplanted through the subcutaneous vein of the forearm.

1.5 Histology, Immunohistochemistry staining evaluation

The experimental animals were sacrificed at day 14, and tissue and blood samples were collected for analysis. Liver tissues were collected immediately after death, washed with normal saline to remove excess blood, and cut into small pieces that could be easily embedded using sterilized surgical scissors and placed in 4 %PFA for fixation. In addition, fresh livers were immediately snap-frozen in liquid nitrogen and stored in a refrigerator at -80 ° C for subsequent analysis. The liver samples were sent to Xi 'an Eke Biotechnology Co., Ltd. for embedding sections with a thickness of 5 μm.

The HE staining procedure was as follows: paraffin sections were deparaffinized, and the deparaffinized sections were placed in hematoxylin staining solution for 5-10 minutes, followed by thorough rinsing with running water to remove excess dye. At this point the nucleus will be stained blue-purple. The stained sections were rapidly differentiated in 1% ethanol hydrochloride solution for a few seconds until the background faded but the nuclei remained distinctly blue-purple. Next, the sections were blued by immersing in running water containing ammonia until the nuclei appeared blue. The sections were transferred to eosin staining solution for 1-3 min to allow the cytoplasm and other

structures to become pink, after which they were quickly rinsed with running water to remove excess dye. The stained sections were dehydrated, excess xylene was volatilized in a fume hood, and neutral resin was added to seal the sections. The results were observed under a microscope.

The Masson staining procedure was as follows: the deparaffinized sections were immersed in hematoxylin staining solution and stained for 5-10 min to stain the nuclei. Subsequently, the sections were rinsed using running water until there was no excess staining solution in the background. The sections were immersed in phosphotungstic acid staining solution for 5-10 min, and this step stained the connective tissue. After completion of staining, the sections were gently rinsed with distilled water. The sections were transferred to bright green staining solution for 2-5 min to give a green appearance to the collagen fibers. After staining, the sections were rinsed again with distilled water. The sections were stained in eosin solution for 1-3 min to stain the cytoplasm and other cellular structures. After completion of staining, the sections were rinsed with running water to remove excess staining solution. After dehydration, the sections were sealed with neutral resin, and the staining results were observed under a microscope.

The steps of PAS staining were as follows: the dehydrated sections were treated in 0.5% Periodic Acid solution for 5-10 minutes to oxidise the 1, 2-diol groups in the polysaccharides to aldehyde groups, and the sections were thoroughly rinsed with running water for 3-5 minutes, taking care not to destroy tissue integrity during this step. The sections were stained in Schiff reagent for 15 to 30 min, and the aldehyde groups reacted with Schiff reagent to form a fuchsin product. After staining, the sections were rinsed with running water for 5-10 min to remove unreacted Schiff reagent. The sections were stained in hematoxylin solution for 2-5 min to stain the nuclei. After staining, sections were rinsed with running water to remove excess staining solution. The sections are dehydrated, sealed, and examined under a microscope. PAS staining is commonly used to detect structures such as glycogen, mucinous material, and basement membranes, which appear purplish red.

The procedure of immunohistochemistry staining was as follows: dehydrated sections were placed in sodium citrate antigen repair solution (pH 6.0) and heated in a microwave oven for antigen repair, and then the sections were allowed to cool to room temperature. After repair, sections were washed three times for 5 min each with PBST (phosphate Tween 20 buffer). Endogenous peroxidase blocking agent was added dropingly and incubated for 15 min at room temperature. Primary antibodies of 1:100 were added, and the sections were incubated overnight at 4 ℃ by placing them in a wet box to prevent drying.

The next day, the primary antibody was washed off with PBST, reaction enhancing solution was then added and incubated for 20 min at 37 ℃. An appropriate amount of enhanced HRP-conjugated goat anti-mouse/rabbit IgG polymer was added and incubated for 20 min at 37 ℃ Finally, DAB staining and hematoxylin staining were performed, and the slides were sealed with neutral resin after dehydration and transparency.

1.6 RNA Sequencing

Mouse liver on day 7 was collected for total RNA extraction, and the RNA sequencing was performed according to the HiSeq 2500 (Illumina) system. Genes with P value <0.05 and fold change >1.0 were considered significant differential genes.

1.7 Statistical Analysis

SPSS 19.0 (IBM Corporation, Chi-cago, USA) software was used for statistical analysis. All experimental data were expressed as mean±SD, and univariate analysis of variance (ANOVA) was used for the results. A P value < 0.05 was considered statistically significant.
